# CREB3L2 Modulates Nerve Growth Factor-Induced Cell Differentiation

**DOI:** 10.3389/fnmol.2021.650338

**Published:** 2021-08-03

**Authors:** Luciana Sampieri, Macarena Funes Chabán, Pablo Di Giusto, Victoria Rozés-Salvador, Cecilia Alvarez

**Affiliations:** ^1^Centro de Investigaciones en Bioquímica Clínica e Inmunología (CIBICI-CONICET), Córdoba, Argentina; ^2^Departamento de Bioquímica Clínica, Facultad de Ciencias Químicas, Universidad Nacional de Córdoba, Córdoba, Argentina; ^3^Instituto de Investigación Médica Mercedes y Martín Ferreyra, INIMEC-CONICET-Universidad Nacional de Córdoba, Córdoba, Argentina

**Keywords:** neuronal differentiation, Golgi complex, CREB3L2, CREB3, PC12, Rab5

## Abstract

Nerve growth factor (NGF) stimulates numerous cellular physiological processes, including growth, differentiation, and survival, and maintains the phenotype of several neuronal types. Most of these NGF-induced processes require adaptation of the secretory pathway since they involve extensive remodeling of membranes and protein redistribution along newly formed neuritic processes. CREB3 transcription factors have emerged as signaling hubs for the regulation of numerous genes involved in the secretory pathway and Golgi homeostasis, integrating stimuli from multiple sources to control secretion, posttranslational modifications and trafficking of proteins. Although recent studies have focused on their role in the central nervous system, little is known about their participation in cell differentiation. Therefore, we aimed to analyze the expression and signaling mechanism of CREB3 transcription factor family members, using the NGF-induced PC12 cell differentiation model. Results show that NGF treatment causes Golgi enlargement and a parallel increased expression of proteins and mRNAs encoding for proteins required for membrane transport (transport factors). Additionally, a significant increase in CREB3L2 protein and mRNA levels is detected in response to NGF. Both MAPK and cAMP signaling pathways are required for this response. Interestingly, CREB3L2 overexpression hampers the NGF-induced neurite outgrowth while its inhibition enhances the morphological changes driven by NGF. In agreement, CREB3L2 overexpressing cells display higher immunofluorescence intensity of Rab5 GTPase (a negative regulator of PC12 differentiation) than control cells. Also, Rab5 immunofluorescence levels decrease in CREB3L2-depleted cells. Taken together, our findings imply that CREB3L2 is an important downstream effector of NGF-activated pathways, leading to neuronal differentiation.

## Introduction

Members of the CREB3 family of transcription factors (CREB3, CREB3L1, CREB3L2, CREB3L3, and CREB3L4) modulate a broad range of cellular processes. They are critical for development, metabolism, secretion, survival, and cell division, among others, and show clear cell-specific expression patterns (Chan et al., 2011). CREB3 members have been implicated in the ER and Golgi stress responses as regulators of the cell secretory capacity and expression of cell specific cargos (Fox and Andrew, 2015; Sampieri et al., 2019). They are ER-localized transmembrane proteins that, in response to the appropriate stimulus, are transported from the ER to the Golgi, cleaved by S1P and S2P proteases; and the released N-terminal cytosolic domains are translocated to the nucleus to regulate transcription of specific target genes (Fox and Andrew, 2015).

CREB3 transcription factors have been also implicated in cell differentiation processes, such as osteoblast differentiation (Murakami et al., 2009) and during human B-cell transition to antibody secreting cells (Al-Maskari et al., 2018). Furthermore, CREB3, CREB3L1, and CREB3L2 are expressed in different cell types of the central nervous system (CNS), where they perform important functions. For instance, CREB3 and CREB3L1 contribute to neuroendocrine regulation of the hypothalamic/pituitary/adrenal axis by modulating the glucocorticoid receptor activity and the arginine vasopressin gene transcription (Greenwood et al., 2017; Penney et al., 2018). Also, in hippocampal cells, CREB3 regulates gene expression of several components of Golgi outposts and, therefore, their formation (Chung et al., 2017). Furthermore, CREB3L2 levels are positively regulated during oligodendrocyte maturation (He et al., 2016), and, in dorsal root ganglia neurons, the C-terminal domain of CREB3L2 is secreted and promotes axon growth (McCurdy et al., 2019). Interestingly, CREB3 has been identified as an NGF-sensitive transcription factor in a comprehensive time-course microarray study performed in PC12 cells (Dijkmans et al., 2008). Despite the advances in the knowledge of CREB3 factors in the CNS, their participation during neuronal differentiation has been poorly explored. In this work, we aim to study the modifications in organelles and proteins associated with the secretory pathway as well as the response and participation of CREB3 transcription factors in the NGF-induced PC12 cell differentiation model (Greene and Tischler, 1976). In this model, NGF activates the Ras/Raf/MEK/ERK signaling pathway (Cowley et al., 1994), which, in turn, triggers a transcriptional program, leading to upregulation of neuronal genes as well as a neurite outgrowth.

In summary, our data indicate that (a) the differentiation process goes along with an increase in proteins and mRNAs encoding for proteins of membrane trafficking pathways; (b) although CREB3, CREB3L1, and CREB3L2 are co-expressed in PC12 cells, only CREB3L2 increases significantly at an early time point of differentiation (6 h); (c) both ERK and PKA signaling pathways associated with NGF-dependent neuritogenesis regulate CREB3L2 expression; (d) a CREB3L2 knockdown affects Golgi phenotype and neuronal differentiation, acting as a negative regulator of this process; and (e) Rab5 levels change after CREB3L2 inhibition or overexpression. Considering our results and the existing literature, we assume that CREB3L2 is a downstream effector of NGF-activated pathways important to neuronal differentiation of PC12 cells.

## Materials and Methods

### DNA Constructs and Antibodies

A CREB3L2FL construct (cloned in pTRE2hyg plasmid) was a kind gift from Dr. Kazunori Imaizumi (Saito et al., 2014). Rab5WT and Rab5S34N, cloned in pEGFP plasmid (Gomez and Daniotti, 2005), were kindly provided by Dr. Alejandro Vilcaes (Universidad Nacional de Córdoba), and Rab1b WT and Rab1b N121I were available in our laboratory (Slavin et al., 2011). shRNA pGFP-C-shLenti vectors were obtained from OriGene (OriGene Technologies, Inc., Rockville, MD, United States).

The following primary antibodies were used: Rab1b (catalog No. SC-599, Santa Cruz Biotechnology, Santa Cruz, CA, United States), MAP2 (catalog No. M2320, Sigma-Aldrich, St. Louis, MO, United States), KAP1 (catalog No. A300-274A, Bethyl Laboratories, Montgomery, TX, United States), GM130 (catalog No. 610823, BD Biosciences, San José, CA, United States), Calreticulin (catalog No. PA3-900, Thermo Fisher Scientific, Waltham, MA, United States), SRP54 (catalog No. 610941, BD Biosciences, San José, CA, United States), GalNAc-T2 (catalog No. HPA011222, Sigma-Aldrich, St. Louis, MO, United States), rabbit polyclonal antibodies to StarD7 [a gift from Dr. Susana Genti-Raimondi, CIBICI-CONICET, National University of Córdoba, Argentina (Angeletti et al., 2008)], Rab5 (catalog No. 46692, Santa Cruz Biotechnology, Santa Cruz, CA, United States), β-actin (catalog No. A2228, Sigma-Aldrich, St. Louis, MO, United States), α-tubulin (catalog No. T9026, Sigma-Aldrich, St. Louis, MO, United States), and CREB3L2 (catalog No. PA5-40951, Thermo Fisher Scientific, Waltham, MA, United States and catalog No. HPA015534, Atlas Antibodies, Stockholm, Sweden). CREB3L2 antibodies were raised against N-terminal residues.

### Cell Culture and Treatments

The rat pheochromocytoma cell line PC12 [ATCC^®^ CRL-1721^TM^ (Greene and Tischler, 1976)] was grown in a normal growing medium, containing Dulbecco’s modified Eagle’s medium (DMEM), 5% fetal bovine serum, 5% of horse serum, and penicillin-streptomycin (Thermo Fisher Scientific, Waltham, MA, United States). For differentiation, cells were grown in the DMEM medium, containing 1.5% horse serum, 1.5% fetal bovine serum, penicillin-streptomycin, and 100 ng/ml nerve growth factor (NGF, catalog No. B.5017, ENVIGO, Indianapolis, IN, United States) for different periods of time. Alternatively, cells were treated with 15 μM forskolin (FSK, catalog No. ab120058, Abcam, Cambridge, United Kingdom). Differentiation conditions include growth onto poly-L-lysine (catalog No. P8920, Sigma-Aldrich, St. Louis, MO, United States), coated plates and coverslips. A MAPK signaling cascade was blocked with 10 μM U0126 (catalog No. 9903, Cell Signaling Technology, Danvers, MA, United States) for 2 h before NGF addition. For transfection, PC12 cells were transiently transfected with Lipofectamine 2000 (Thermo Fisher Scientific, Waltham, MA, United States), following the instructions of the manufacturer. Complexes containing different DNA constructs plus Lipofectamine 2000 were resuspended in Opti-MEM (Thermo Fisher Scientific, Waltham, MA, United States) and mixed with 10% of fetal bovine serum; after 4 h, the medium was replaced for a normal growing medium. After 24 h of transfection, cells were NGF differentiated. For shRNA experiments, transfections were performed for 48 h before NGF differentiation.

### Immunofluorescence Analysis

Cells grown on poly-L-lysine (catalog No. P8920, Sigma-Aldrich, St. Louis, MO, United States) were fixed, blocked, and immunolabeled as described previously (Gil et al., 2004). Primary antibodies were diluted as follows: anti-GM130 at 1:200; anti-CREB3L2 at 1:200; anti-Rab5 at 1:75. Secondary antibodies were diluted at 1:1000. Nuclei were stained, using Hoechst 33258 (catalog No. H-3569, Molecular Probes, Eugene, OR, United States) at 1:1000.

### Image Acquisition and Quantification

Image acquisition was performed for 2D images, using the Leica DMi8 epifluorescence microscope (lasers: 488; resolution *X* = 1024 and *Y* = 1024; objectives: 40× and 63×) and for 3D images, using either a spectral (Olympus Fluoview 1200) or LSM 800 (Zeiss) (lasers: 488, 533, and 633; resolution *X* = 1024; *Y* = 1024 and *Z* = 0.3–0.5 μm; objectives: 63×: plan-apochromat 63×/1.40 Oil DICM27 and 20×: objective 20× LD apochromat 20×/0.40, both inverted confocal microscopes). Image quantification was performed, using Fiji-ImageJ software (Schindelin et al., 2012), pixel by pixel, and data were used to calculate the average of Golgi volume. To quantify GM130, GalNAc-T2, CREB3L2, and Rab5 levels, total fluorescence intensity was calculated throughout the *z*-axis, using the “z-project/sum slices” plug-in of Fiji-ImageJ. Then, the soma was measured as previously described (Siri et al., 2020). The results were normalized with the control condition of each experiment. Fire-LUTs are shown to clearly visualize the fluorescence levels of each epitope.

### Morphometric Analysis

Images of differentiated PC12 cells were processed, using a Fiji-ImageJ software macro. After image processing, total neurite length and the longest neurite of each cell were measured. Sholl analysis was performed to quantify the number of intersections in the neurite outgrowth of the cells [Sholl analysis v3.4.10 plug-in for ImageJ (Sholl, 1953)]. For measurements, a straight line was traced from the center of the cell body to the end of the neurites; intersections were analyzed, defining five shells, starting at 30 μm of the cell soma to the last shell at 150 μm.

### Protein Analysis

Cell processing and Western blot assays were performed as described previously (Garcia et al., 2017). Detection and quantification of the near-infrared fluorescence on the membranes were performed, using the Odyssey CLx Imaging System (LI-COR Biosciences, Lincoln, NE, United States) through the Image Studio Software. Images were acquired on the auto intensity at high resolution. The following primary antibody dilutions were used: anti-MAP2 at 1:1000, anti-KAP1 at 1:1000, anti-SRP54 at 1:500, anti-GalNAc-T2 at 1:500, anti-StarD7 at 1:500, anti-Rab1b at 1:100; anti-GM130 at 1:400; anti-CREB3L2 at 1:500; anti-calreticulin at 1:2000; anti-α-tubulin at 1:2000 and anti-β-actin at 1:1000.

For Western blot quantification, the intensity of each band normalized to β-actin or to KAP1 (loading controls) was measured, and the fold change was calculated as the ratio of the normalized values in the differentiated (D2 to D6) versus control cells (D0). Unless indicated otherwise, three independent experiments were performed, and each sample was run in duplicate. The normalized value of one control was set as one, the other values of the controls were calculated relative to these values, and average values are shown in the bar graphs. Therefore, the values in the different control conditions are close to one, and their error bars represent ± SEM.

### RNA Isolation and RT-qPCR

Total RNA was purified from PC12 cells by using TRI Reagent (catalog No. T9424, Sigma-Aldrich, St. Louis, MO, United States) according to the protocol of the manufacturer. Synthesis of cDNA was performed from 1 μg of total RNA in a total volume of 20 μl, using random primers (catalog No. C118A, Promega, Madison, WI, United States) and 50 U M-MLV reverse transcriptase (catalog No. M1705, Promega, Madison, WI, United States). Primers were designed with the assistance of the NetPrimer software (PREMIER Biosoft International, Palo Alto, CA, United States). Primers were from Sigma-Aldrich (Houston, TX, United States) or Macrogen (Seoul, South Korea), and their concentrations and sequences (5′–3′) are: Sec31a (150 nM), ATTCGGAGGGAAGTTGGTGAC (F), TCTGAGC GGCTGAGGAAGTC (R); GM130 (150 nM), CGGGA TGTCGGAAGAAAC (F), GTGTGGTCTGTGGGCACATT (R), Rab1b (250 nM) AACGGTTCAGGACCATCACTTC (F) TCTCACTGGCGTAGCGATCTATT (R); KDELR3 (100 nM), GGCATCTCTGGGAAGAGTCAG (F), ATAGGCACACAGGA GGAAAACC (R); CREB3L1 (300 nM), GTGAAAGA AGACCCCGTCGC (F), CTCCACAGGCAGTAGAGCACC (R); CREB3L2 (300 nm), CGGGCTCAGTCACCATTTACC (F), CCATTTCTCACTCTCCACCTCC (R); CREB3 (200 nM), GGAAAGTGGAGATTTGTGGGC (F), GCACGGAGTTCTCG GAAG (R); Rab7a (100 nM forward primer and 75 nM reverse primer), GGAGGTGATGGTGGATGACAG (F), GG GTTTTGAATGTGTTGGGG (R), Rab5a (250 nM forward primer and 150 nM reverse primer), TTCTTCTAGGAGA GTCTGCTGTTGG (F), CATCAAGACACACAGTTTGGGTT (R), MAP2 (300 nM), GACGGACCACCAGGTCAGAA (F), ACGTGAAGAGTAGCTTGGAGGAGT (R), TBP (300 nM), GCACAGGAGCCAAGAGTGAA (F), CACATCACAGCTCCC CACC (R). qPCR analysis was performed, using an ABI Prism 7500 detection system (Applied Biosystems, Foster City, CA, United States) and SYBR Green chemistry. Reactions were carried out in triplicate, using 1X SYBR Green PCR Master Mix (catalog No. 4309155, Thermo Fisher Scientific, Waltham, MA, United States) in a total volume of 15 μl. Specificity was verified by melting curve analysis and agarose gel electrophoresis. The fold change in gene expression was calculated according to the 2^–ΔΔCt^ method, using TBP as the internal control (Livak and Schmittgen, 2001).

### shRNA

CREB3L2 expression was inhibited by using a commercial pEGFP-C-shLenti vector (OriGene Technologies, Inc., Rockville, MD, United States). This vector was designed to specifically inhibit CREB3L2 expression (shCREB3L2 I: 5′-AAC CTCAAGGTTGTAGAACTGGAGAGGA-3′; shCREB3L2 II: 5′- AGCACCTCTCATCCAGGCTGAACACAGCT-3′) and encodes GFP protein as a marker of transfection. Lentiviral particles were obtained by co-transfecting the HEK293T cell line with pEGFP-C-shLenti, psPAX2, and pMD2.G plasmids. PC12 cells were then transduced, and, after 3 days of puromycin selection, quantification of neurite length and number, following NGF-differentiation, was carried out. Lentiviral particles expressing scrambled shRNA (shScramble: 5′-GCACTACCAGAGCTAACTCAGATAGTACT-3′) were used as control.

### Statistical Analysis

Results are presented as the mean ± SEM of at least three independent experiments performed in duplicates or triplicates. Comparisons between two groups were made by using an unpaired Student’s *T*-test. Multiple group analysis was conducted by one-way ANOVA. As a post-test, the Bonferroni multiple-comparison test was used. Statistical analysis was performed by using GraphPad Prism 5.0 software (GraphPad Software, San Diego, CA, United States). Differences were considered significant at *p* < 0.05.

## Results

### NGF-Induced Cell Differentiation Promotes Changes in the Secretory Pathway

**FIGURE 1:**
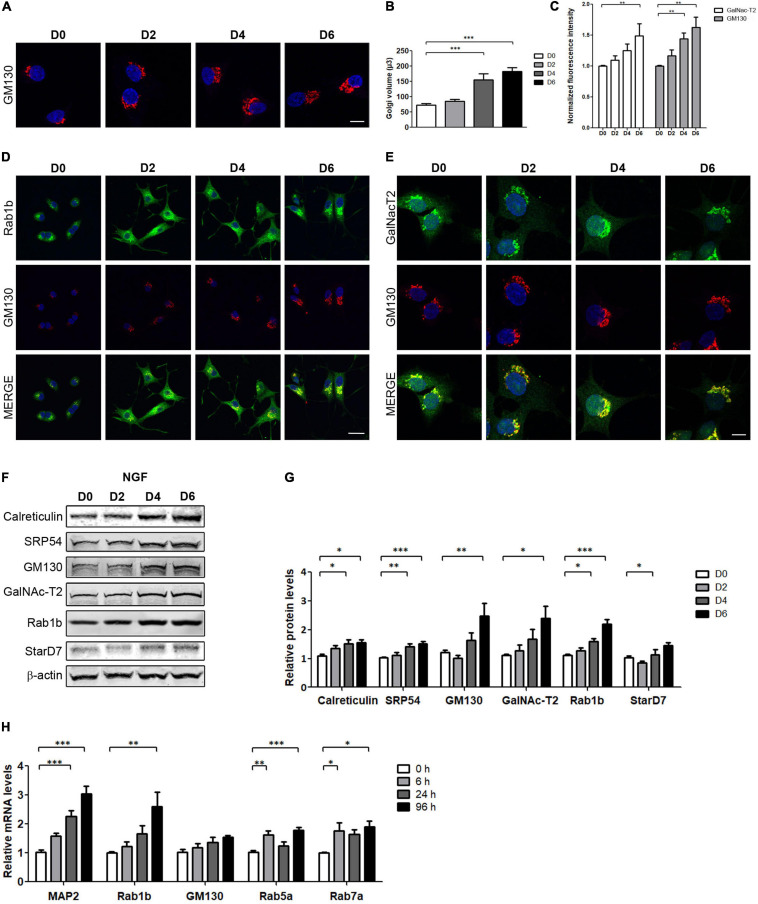
NGF-induced PC12 cell differentiation increases Golgi volume and transport proteins. **(A,D,E)** Immunofluorescence performed in PC12 cells differentiated with NGF (100 ng/ml) during the indicated days and stained with the specified markers. Nuclei were stained with Hoechst (blue). **(A,C)** The Golgi complex was labeled with GM130 (red, Bar: 8 μm) and the normalized fluorescence intensity was plotted (a total of 60 cells were analyzed). **(B)** Golgi volume was quantified on the indicated days. Fiji-ImageJ software was used to perform three-dimensional reconstruction and quantification of the images. **(D,E)** Rab1b, GM130, and GalNAc-T2 staining. Scale bars: 20 and 8 μm, respectively. **(F)** Representative Western blot assays performed with cell lysates obtained from PC12 cells differentiated with NGF during the indicated days (D2, D4, and D6). **(G)** Densitometric quantification of proteins shown in **(F)** normalized to β-actin. Values represent fold change relative to protein levels in untreated cells (D0). **(H)** Quantification of the indicated genes by qRT-PCR performed with total RNA during the indicated times. Results were normalized to the levels of TBP and expressed according to the 2^–ΔΔCt^ method relative to the expression level of each gene in untreated cells (0 h, set as 1). Bars represent the mean ± SEM of three independent experiments carried out in triplicates. **(B,C,G,H)** Statistical data analysis were performed using ANOVA test, followed by Bonferroni multiple comparison post-test, considering statistically significant a value of *p* < 0.05 (^∗^*p* < 0.05; ^∗∗^*p* < 0.001; ^∗∗∗^*p* < 0.0001).

To investigate changes in the secretory pathway during NGF-induced PC12 cell differentiation, cells were treated with NGF during 6 days as previously reported (Galbiati et al., 1998; Vanhoutte et al., 2001). The differentiated phenotype was determined by analyzing the neurite outgrowth, morphological transformations, as well as the changes in the expression levels of the neuron-specific protein MAP2 (∼8-fold relative to the control, Supplementary Figures 1A,B). Next, we evaluated the effect of NGF induction on Golgi morphology by the immunostaining of *cis*-Golgi marker GM130, using confocal microscopy and acquiring Z-stack images. As shown in Figures 1A,C, the fluorescence intensity of GM130 signal increases in response to NGF, being evidently visible between days 4 and 6. Thus, to further analyze changes induced by NGF differentiation, Golgi volume was assessed by 3D reconstruction of Z-stacks, and a set of transport proteins was analyzed. In agreement with the increase of fluorescence intensity, Golgi volume increases ∼1.5- to 2-fold after 4 and 6 days of NGF treatment (Figure 1B). Moreover, we analyzed the fluorescence intensity of other proteins associated with membrane trafficking, such as the GTPase Rab1b, essential for ER to Golgi transport, localized at the ER-Golgi-Intermediate compartment (Garcia et al., 2011; Slavin et al., 2011) and the *medial*- and *trans*-Golgi marker GalNAc-T2 (Rottger et al., 1998). As shown in Figures 1C–E, the fluorescence intensity of both protein markers correlates with the increase of the GM130 signal. In agreement with the confocal examination, Western blot analysis (Figures 1F,G) indicated that GM130, GalNAc-T2, and Rab1b levels increased more than 2 times relative to untreated cells (D0). Additionally, the expression levels of the ER markers calreticulin and SRP54, and of the phosphatidylcholine transfer protein StarD7 [one of the main lipid transfer proteins associated with membrane expansion in NGF-treatment of PC12 cells (Durand et al., 2004; Yang et al., 2017)] increase up to 1.5 times relative to control. To verify that increased protein levels are associated with transcriptional changes, quantitative real-time reverse transcription-polymerase chain reaction assays (qRT-PCR) were performed at different times after NGF treatment (Figure 1H), and the relative mRNA levels of undifferentiated (0 h) and NGF-differentiated PC12 cells (6, 24, and 96 h) were evaluated. We analyzed MAP2 transcript levels as a positive control of NGF treatment (Fischer et al., 1991), as well as mRNAs encoding for Rab1b, GM130, and two endocytic pathway proteins, Rab5 and Rab7. NGF induced a progressive increase in MAP2 and Rab1b mRNA levels during the analyzed time, reaching 3-fold and ∼2.5-fold increase relative to the control for 96 h. GM130 displays a slight increase after 6 h of NFG treatment and remains stable throughout the analyzed times. Rab5a and Rab7 also increase significantly during differentiation with NGF (Figure 1H). Taken together, these results suggest that the homeostatic cellular response to NGF involves transcriptional regulation of multiple genes-encoding proteins of different compartments of the membrane trafficking pathway.

### NGF-Induced PC12 Differentiation Strongly Increases CREB3L2 Expression

**FIGURE 2:**
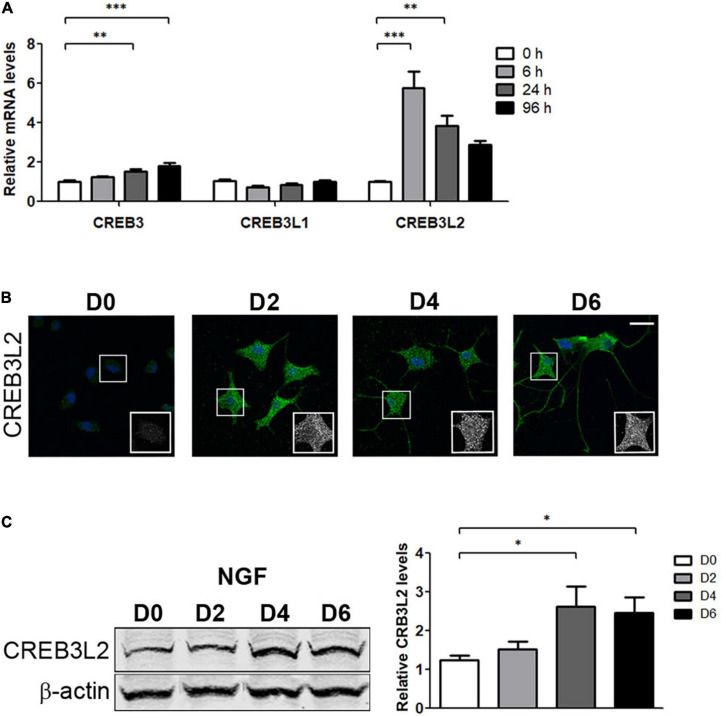
Effect of NGF on CREB3 transcription factor expression. PC12 cells differentiated with NGF at the indicated times. **(A)** Quantification of CREB3, CREB3L1, and CREB3L2 mRNA levels by qRT-PCR performed with total RNA. The results were normalized to the levels of TBP and expressed according to the 2^–ΔΔCt^ method relative to the expression level of each gene in untreated cells (0 h, set as 1). **(B)** Representative immunofluorescence staining of CREB3L2 in PC12 cells at the indicated times of NGF incubation. Nuclei were stained with Hoechst (blue). Insets show the ROI (white square) magnification from each image. Scale bar: 20 μm. **(C)** Left panel: Representative Western blot assays (detecting the CREB3L2 N-terminal domain or cleaved fragment) performed with cell extracts obtained from PC12 cells treated with NGF during the indicated times. Right panel: Densitometric quantification of CREB3L2 protein shown in left normalized to β-actin. Values represent fold change relative to protein levels in untreated cells (D0). Bars represent the mean ± SEM of three independent experiments carried out in triplicates. Statistical data analysis was performed using ANOVA test, followed by Bonferroni multiple comparison post-test, considering statistically significant a value of *p* < 0.05 (^∗^*p* < 0.05; ^∗∗^*p* < 0.001; ^∗∗∗^*p* < 0.0001).

CREB3 factors have been characterized as regulators of genes-encoding components of the secretory pathway (Fox et al., 2010; Sampieri et al., 2019). In addition, CREB3, CREB3L1, and CREB3L2 are expressed in different cell types of the CNS (MacGillavry et al., 2011; Okuda et al., 2014; Ying et al., 2014; Sumida et al., 2018). We hypothesize that levels of the CREB3 family members could be modified during PC12 cell differentiation, and, therefore, mRNA levels of all were quantified by qRT-PCR assays (Figure 2A). The results indicated that CREB3 levels slightly increased ∼1.5- and 1.8-fold after 24 and 96 h of NGF treatment, respectively. On the other hand, CREB3L1 levels remained constant during the evaluated times, and CREB3L2 levels increased rapidly and noticeably at 6 h after NGF treatment (∼6-fold), followed by a gradual decline after 24 and 96 h of NGF treatment. Interestingly, the response of CREB3L2 to NGF is fast, at 6 h, whereas CREB3 does not show a significant increase at the same time. Furthermore, at later times (24 and 96 h), CREB3 mRNA levels slightly increase, while those of CREB3L2 decrease, suggesting that CREB3L2 leads an early homeostatic cellular response. In contrast to CREB3, CREB3L1 and CREB3L2, the transcript levels of CREB3L3 and CREB3L4 were extremely low, making it difficult to quantify their changes during differentiation (data not shown). Additionally, we analyzed the CREB3L2 protein expression changes by immunofluorescence and Western blot assays. As shown in Figures 2B,C, the immunofluorescence signal of CREB3L2 increased during NGF differentiation: full length CREB3L2 is localized at the ER as indicated by its reticular pattern, and the N-terminal domain (or cleaved fraction) colocalizes with Hoechst in the nucleus (Murakami et al., 2009; Saito et al., 2014). Taken together, these findings indicate that NGF induces an important upregulation of CREB3L2 expression.

### CREB3L2 Expression Requires MAPK and cAMP Signaling Pathways

**FIGURE 3:**
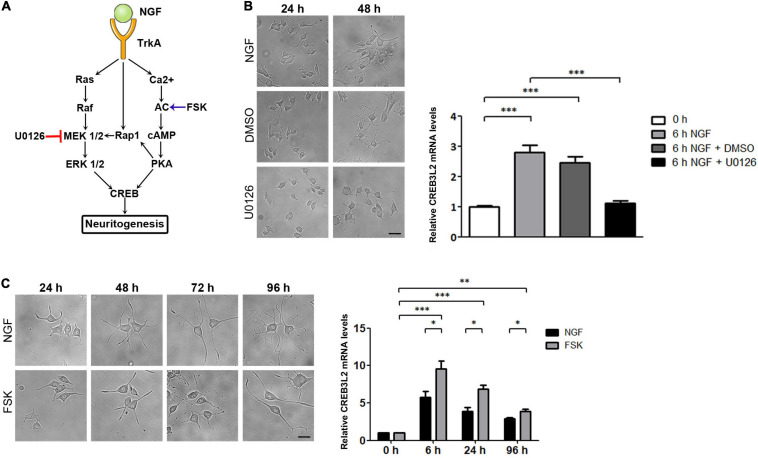
Effect of U0126 and Forskolin on neuritogenesis and CREB3L2 expression. PC12 cells differentiated with NGF during the indicated times in the presence of vehicle (DMSO), U0126 (10 μM), or Forskolin (FSK, 15 μM). DMSO and U0126 were added 2 h before NGF induction. **(A)** Schematic representation of NGF-activated signaling pathways in PC12 cells with the site of action of U0126 and FSK. **(B)** Left panel: Representative images of cells incubated with NGF, or with NGF and either DMSO or the MAPK/ERK 1/2 inhibitor, U0126, during the indicated times. Scale bar: 10 μm. Right panel: qRT-PCR of CREB3L2 transcript levels performed with total RNA extracted from cells incubated with NGF at the indicated times. **(C)** Left panel: Representative images of cells incubated with NGF or Forskolin during the indicated times. Scale bar: 10 μm. Right panel: qRT-PCR of CREB3L2 transcript levels performed with total RNA extracted from cells incubated with Forskolin; mRNA values **(B,C)** were normalized to the levels of TBP and expressed according to the 2^–ΔΔCt^ method relative to the expression level of CREB3L2 in untreated cells (0 h, set as 1). Bars represent the mean ± SEM of three independent experiments carried out in triplicates. Statistical data analysis was performed using ANOVA test followed by Bonferroni multiple comparison post-test, considering statistically significant a value of *p* < 0.05 (^∗∗^*p* < 0.001, ^∗∗∗^*p* < 0.0001).

It has been shown that PC12 differentiation induced by NGF is mediated by its interaction with the tyrosine kinase receptor type 1, TrkA (Kaplan et al., 1991; Klein et al., 1991). Upon interaction with NGF, TrkA is phosphorylated and triggers the Ras/Raf/MEK/ERK signaling pathway to stimulate CREB phosphorylation. Furthermore, in PC12 cells, the second messenger cyclic AMP (cAMP) also mediates the action of NGF in a calcium-dependent manner (Stessin et al., 2006) *via* the PKA-CREB-dependent signaling pathway (Figure 3A).

To determine whether the Ras/Raf/MEK/Erk pathway was associated with the effect of NGF on CREB3L2 expression, CREB3L2 mRNA levels were evaluated after treatment with U0126 (Figure 3A), a pharmacological inhibitor of MAP kinases MEK1 and MEK2 (Duncia et al., 1998). As shown in Figure 3B, the inhibitor was effective and blocked differentiation even after 48 h of NGF incubation. Also, quantification of CREB3L2 mRNA performed after 6 h of NGF addition indicates that U0126 inhibited the increase of mRNA CREB3L2 levels induced by NGF.

To analyze the influence of the cAMP pathway in the NGF-induced increase of CREB3L2, the effect of the adenylate cyclase activator forskolin (FSK, Figure 3A) was tested (Seamon et al., 1981). PC12 cells were incubated with FSK (15 μM), and as performed with U0126, its effect on the neurite outgrowth was first confirmed microscopically (Figure 3C). As previously shown (Richter-Landsberg and Jastorff, 1986), the increase in the number and length of neurites in response to FSK was similar or slightly lower to that induced by NGF. FSK also increased CREB3L2 mRNA levels with the same kinetics as NGF, reaching a maximum value at 6 h after treatment (Figure 3C). Remarkably, CREB3L2 transcript levels increased up to 10 times at 6 h of FSK treatment relative to the untreated cells (0 h), whereas the maximum CREB3L2 mRNA increase after 6 h of NGF addition was 6 times with respect to untreated cells.

Taken together, the data indicate that both MAPK and cAMP signaling pathways are associated with neurite formation as well as with the upregulation of CREB3L2 expression and highlight a key role of CREB3L2 as a common effector of ERK and cAMP pathways.

### CREB3L2 shRNA-Mediated Knockdown and CREB3L2 Overexpression Modify the NGF-Induced Neuronal Differentiation Phenotype of PC12 Cells and Rab5 Protein Levels

**FIGURE 4:**
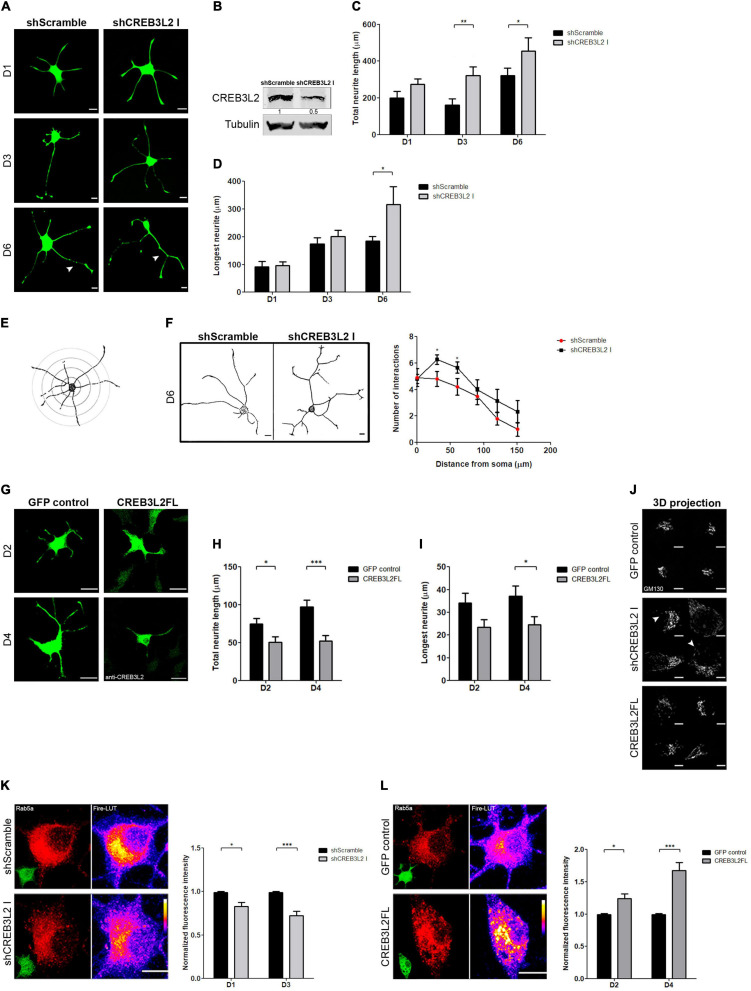
The suppression of CREB3L2 alters the correct neuronal differentiated phenotype of PC12 cells. **(A)** Representative images of differentiated PC12 cells transduced with shScramble-EGFP (shScramble) or shCREB3L2-EGFP (shCREB3L2 I) and fixed at the indicated time after NGF induction. Arrowheads indicate the longest neurite. **(B)** Representative Western blot assays performed with cell lysates obtained from differentiated PC12 cells transduced with shScramble or shCREB3L2 I and blotted against CREB3L2 (detecting the N-terminal domain or cleaved fragment) and α-tubulin. Quantification of the total neurite length **(C)** and the longest neurite **(D)**. **(E)** Schematic representation of the Sholl analysis performed in the cells. **(F)** Left panel: Representative images of differentiated PC12 cells transduced with shScramble or shCREB3L2 I and fixed at 6 days post NGF induction. Acquired images were inverted for morphometric analysis. Right panel: Quantification of the number of intersections crossing the shells. **(G)** Representative images of differentiated PC12 cells transfected with control EGFP or CREB3L2FL plasmids and fixed at 2 and 4 days post NGF induction. Quantification of the total neurite length **(H)** and the longest neurite **(I)**. **(J)** 3D projection of GM130 immunostaining in cells transduced with shCREB3L2 I or transfected with control EGFP or CREB3L2 FL plasmids. Arrowheads indicate punctate structures labeled with GM130. **(K)** Left panel: Representative images of differentiated PC12 cells transfected with shScramble or shCREB3L2 I and fixed at 3 days post NGF induction and immunostained for Rab5. Fire-LUTs are shown to clearly visualize IF levels. Right panel: Quantification of Rab5 fluorescence intensity normalized with the control. **(L)** Left panel: Representative images of differentiated PC12 cells transfected with control-EGFP or CREB3L2 FL plasmids and fixed at 4 days post NGF induction and immunostained for Rab5. Fire-LUTs are shown to clearly visualize IF levels. Right panel: Quantification of Rab5 fluorescence intensity normalized with the control. Bars represent the mean ± SEM of three independent experiments carried out in triplicates, and a total of 50 cells were analyzed. Statistical data analysis was performed, using the Student’s *T*-test, considering statistically significant a value of *p* < 0.05 (^∗^*p* < 0.05, ^∗∗^*p* < 0.01; ^∗∗∗^*p* < 0.001). Scale bars **(A,G)** 20 μm; **(J,K,L)** 5 μm.

To analyze the role of CREB3L2 during PC12 cells differentiation, loss- and gain-of-function experiments were performed. Loss of function was achieved by using a specific shRNA sequence to selectively decrease CREB3L2 expression (see section “Materials and Methods”). PC12 cells were transduced with lentiviral particles-encoding shRNA against CREB3L2 (shCREB3L2 I) or a non-specific shRNA used as control (shScramble), and after puromycin selection, cells were differentiated with NGF (Figure 4A). CREB3L2 levels in cells expressing shCREB3L2 were ∼50% less than in control cells (Figure 4B). Total neurite length and extension of the longest neurite were quantified at days 1, 3, and 6 post-NGF addition in both shCREB3L2 and control-transduced cells (Figures 4C,D). The results indicated that total neurite length increased at D3 and D6 of NGF treatment in shCREB3L2-transduced cells relative to control cells. Likewise, a significant increase in the longest neurite length was observed at D6 in shCREB3L2-treated cells relative to control cells. Similar results were also observed in cells transiently transfected with an shRNA (named shCREB3L2 II), targeting a different sequence of CREB3L2, arguing against off-target effects (Supplementary Figure 2). The data indicate that CREB3L2 inhibition deregulates the neurite outgrowth during the neuronal differentiation process of PC12 cells. Furthermore, Sholl analysis was performed to analyze morphology of the neurite outgrowth (Sholl, 1953; Brown et al., 2011). To this end, five concentric shells spaced every 30 μm each were defined around the cell soma, and the number of intersections crossing the shells was quantified (Figure 4E). Cells expressing shCREB3L2 showed more intersections at 60 and 90 μm from the soma than control cells (Figure 4F). Moreover, overexpression of CREB3L2FL induced a decrease in the total neurite length and the longest neurite as well (Figures 4G–I). Furthermore, in shCREB3L2-treated cells, punctate structures labeled with GM130 were detected throughout the soma of the cells (Figure 4J), suggesting that CREB3L2 depletion induces Golgi fragmentation. Although some GM130-punctated structures were also observed in CREB3L2 overexpressing cells, this phenotype was stronger in CREB3L2 knockdown cells (Figure 4J).

These results were puzzling and contradicted our initial predictions. If CREB3L2 increases during NGF-induced differentiation, how does its knockdown promote a neurite outgrowth and its overexpression inhibit the same process? Are these phenotypes related to the activity of membrane trafficking pathway-related proteins? To address these questions, we evaluated PC12 differentiation after interfering with ER-Golgi or endosomal transport by over-expressing Rab1b or Rab5 constructs, respectively (Plutner et al., 1991; Bucci et al., 1992). PC12 cells were transiently transfected for 24 h and then treated with NGF for 2 and 4 days. Interestingly, overexpression of wild type Rab1b (Rab1b WT) induced the neurite outgrowth, while the dominant negative construct, Rab1b N121I (Pind et al., 1994), inhibited it (Supplementary Figure 3). These effects were opposite to those observed by CREB3L2 overexpression or inhibition, respectively. In contrast, the neurite outgrowth was impaired by overexpression of wild-type Rab5, whereas it was promoted by the dominant negative Rab5 construct, Rab5 S34N (Supplementary Figure 3; Liu et al., 2007). Rab5 phenotypes were similar to those observed after CREB3L2 inhibition and overexpression, which prompted us to examine whether CREB3L2 affects Rab5 expression. To that end, cells were transiently transfected with shScramble, shCREB3L2, pEGFP or CREB3L2FL, and the Rab5 fluorescence signal was quantified (an anti-CREB3L2 antibody was used to detect CREB3L2 overexpressing cells). Immunofluorescence analysis revealed decreased Rab5 levels in shCREB3L2-transfected cells compared with control cells (scramble shRNA) after NGF differentiation. In contrast, in CREB3L2 overexpressing cells, Rab5 fluorescence intensity was higher than in control cells (Figures 4K,L). Taken together, the data indicate that CREB3L2 modulates NGF-induced cell differentiation and strongly suggest that Rab5 GTPase is one of the CREB3L2 targets.

## Discussion

Several studies carried out in different polarized cell types have shown that the membrane trafficking pathway provides membranes needed to achieve cell polarization (Lecuit and Wieschaus, 2000; Ye et al., 2006). PC12 cells, when cultured in the absence of the nerve growth factor, are small round (about 10 μm in diameter) or polygonal-shaped cells and have very few if any neurite-like processes. NGF treatment induces a dramatic increase in cell size (Seaborn et al., 2014; Supplementary Figure 1). Consistent with these data, Martorana et al. (2018) have shown that NGF differentiation goes along with increased mitochondrial remodeling, involving higher levels of fission and fusion proteins. To accomplish differentiation, a massive expansion of the cell membrane occurs, and this event should be accompanied by an increase in lipid content. Several reports have already shown that NGF-induced differentiation implies higher lipid synthesis (Araki and Wurtman, 1997; Li and Wurtman, 1998), particularly of phosphatidylcholine. In this regard, an increase in StarD7, a protein implicated in the delivery of phosphatidylcholine to mitochondria, was detected in the PC12 cells after NGF addition (Figure 1F).

Neurons display a perinuclear or cell body-localized Golgi apparatus and small Golgi cisternae located in dendritic spines called “Golgi outposts” [GOPs, (Horton et al., 2005)]. The size and the number of GOPs are related to the differentiation stage and function of the neurons. In fact, Golgi volume and dendritic GOPs increase during neuronal development (Horton et al., 2005). In agreement with this, our results show that the differentiation of PC12 cells induced by NGF is accompanied by an increase of both the volume of the Golgi complex and the expression levels of proteins associated with the secretory pathway (Figure 1). Transcripts encoding for transport factors also increased. This suggests that the differentiation program induced by NGF not only induces transcription of neuronal-specific genes associated with the differentiated phenotype but also genes that encode proteins ubiquitously expressed involved in the cellular homeostatic process elicited during PC12 differentiation. Furthermore, mRNA levels analysis of the CREB3 family members showed that CREB3, CREB3L1, and CREB3L2 are co-expressed in PC12 cells. However, only CREB3L2 mRNA levels significantly increase after 6 h of NGF induction, whereas CREB3 slightly increases only after 24 h, suggesting that CREB3L2 is a member of the CREB3 family that acts as an early mediator of NGF-induced differentiation (Figure 2). Interestingly, CREB3L2 mRNA levels increase out of a phase with the increase of the cleaved CREB3L2 fraction (Figures 2A,C). Also, immunofluorescence assays reveal an increase in full-length CREB3L2 (represented by the ER pattern, Figure 2B). These results clearly show that CREB3L2 regulation is a complex process, involving both transcriptional and posttranslational regulation (through S1P and S2P proteases).

Differentiation in neuronal cells can be induced by an increase in intracellular concentrations of cyclic AMP (cAMP) or calcium (Sanchez et al., 2004). cAMP and calcium, in turn, activate specific signaling pathways, which ultimately lead to the activation of transcription factors and genes involved in the differentiation program. In NGF-treated PC12 cells, activation of the ERK/MAPK signaling pathway leads to the induction of a set of genes called “immediate-early genes” [IEGs, (Sheng and Greenberg, 1990)]. IEGs are activated in a rapid, robust, and transient manner, and independently of new protein synthesis (Greenberg et al., 1985). Many of these IEGs (as *c-fos*) are transcription factors required for the activation of a second set of genes-encoding proteins that may contribute to the differentiation process (Sheng and Greenberg, 1990). In PC12 cells, c-Fos is necessary for neurite elongation (Gil et al., 2004), and its mRNA levels increase as early as 15 min after NGF addition (Milbrandt, 1986). Our observations that CREB3L2 mRNA levels increase 6 h after NGF addition suggest that, in a similar manner to that of *c-fos*, the rapid response of CREB3L2 may be required during the first stages of NGF-induced differentiation. PKA and MAPK/ERK are two extensively studied signaling pathways that lead to neuritogenesis. According to our results, CREB3L2 is a downstream effector shared between these two signaling pathways (Figure 3).

The acquisition of the differentiated phenotype and neurite growth is accomplished by several cellular adaptations, such as membrane addition, redistribution of molecules and organelles, cytoskeleton regulators, and the activation of their associated proteins (Horton and Ehlers, 2003; Neukirchen and Bradke, 2011; Takano et al., 2015). Some of the regulators of these events are proteins associated with the membrane trafficking pathway. In this regard, our results suggest that CREB3L2 acts as a negative regulator of neuritic outgrowth (Figure 4). Interestingly, the suppression of TBC1D12 (a recycling endosome-resident protein) promotes neurite development in differentiated PC12 cells (Oguchi et al., 2017). Similar results were observed in a neuroblastoma cell line with the expression of p160ROCK [a Rho-associated protein kinase associated with microtubules dynamics (Hirose et al., 1998)]. In addition, a knockout of the transcription factor KLF4 enhances axon and neurite growth in retinal ganglion cells (Moore et al., 2009).

Changes in CREB3 agree with those observed in a microarray study performed on PC12 cells treated with NGF (Dijkmans et al., 2008). Although it has been described that CREB3 participates in axonal regeneration (Ying et al., 2014, 2015) and that, in astrocytes, CREB3L1 impedes axon growth and functional recovery after spinal injury (Sumida et al., 2018), many questions about the molecular mechanisms of CREB3 transcription factors in these neuronal processes remain unknown. In terms of the participation of CREB3 transcription factors in differentiation processes, our research group (Garcia et al., 2017) has previously reported the adaptation of the Golgi complex in a CREB3L1-dependent manner in rat thyroid cells incubated with TSH (thyroid stimulating hormone). It has been demonstrated that, during chondroblasts differentiation into mature chondrocytes, CREB3L2 mRNA synthesis and proteolytic activation are induced (Saito et al., 2009). Work by Al-Maskari et al. (2018) shows that, during B-cell differentiation into antibody-secreting plasma cells, not only do CREB3L2 levels increase but also its processing. Moreover, blocking S1P-mediated proteolysis prevents activated B-cells from becoming antibody-secreting cells (Al-Maskari et al., 2018).

Furthermore, morphometric analysis shows that cells expressing shCREB3L2 display a more complex neuritic outgrowth than the control cells (Figure 4). This can be easily linked to previous reports where the modulation of both endocytic and secretory proteins affects the complexity of the neuritic outgrowth. For example, inhibition of the small GTPase Rab11 increases dendritic arborization in hippocampal neurons (Siri et al., 2020). Also, Rab35-suppression enhances the number of neuritic processes and acts as a negative regulator during differentiation of the oligodendroglial progenitor cell line (Miyamoto et al., 2014), and the knockdown of Rab5a and b, Rab20, and Rab32 in PC12 cells promotes the neurite outgrowth (Oguchi et al., 2018). Regarding Rab5a and Rab32, *in silico* analysis reveals putative CREB3L2 response elements in their promoter regions (data not shown). In agreement with this, CREB3L2 inhibition and overexpression decreased and increased the expression of Rab5, respectively (Figure 4). Rab5 is a central player in the NGF-TrkA signaling pathway required for PC12 differentiation. Its activity impairs the correct neurite outgrowth through the inactivation of TrkA signaling by promoting fusion of early endosomes. This is a necessary step for their transition to late endosomes and subsequent lysosomal degradation (Rink et al., 2005; Liu et al., 2007). Thus, the effect of CREB3L2 on the neurite outgrowth strongly suggests that it regulates Rab5 levels. This is consistent with previous evidence about the inhibitory role of Rab5 in PC12 differentiation (Liu et al., 2007) and explains the negative regulatory effect of CREB3L2 in the NGF-induced differentiation process. Moreover, Golgi fragmentation due to CREB3L2 downregulation also reflects a correlation between CREB3L2 function and membrane trafficking. Although Golgi fragmentation can be a common consequence of various processes involving cellular stress; it is also an indicator of disturbances at vesicular transport levels (Makhoul et al., 2018), suggesting that CREB3L2 could regulate the expression of other molecules involved in different steps of membrane trafficking. In this sense, the partial downregulation of CREB3L2 achieved with both shRNA sequences used could explain the mild effect observed on the Golgi phenotype. However, we cannot exclude any option, nor that it may be due to the redundancy of some functions between CREB3L2 and CREB3 or CREB3L1, or that they have completely different target genes. Our data also show that altering ER to Golgi transport, by regulating Rab1b levels or activity, affects PC12 differentiation (Supplementary Figure 3). In agreement, the importance of Rab1 levels and ER-Golgi transport has been previously characterized in different cellular models (Cooper et al., 2006; Tomas et al., 2012; Romero et al., 2013). Additional studies must be performed to determine the mechanisms that regulate the expression of proteins involved in different membrane transport steps. Neuronal differentiation is a remarkable process, consisting of fine tuning of inhibitory and stimulatory events exerted by a large number of proteins. Disruption of this intricate network at any level is expected to alter the outcome of the differentiating cell program. On the other hand, such a complex process does not rely on a single protein to carry out one unique function, which is why proteins belonging to the same family can be found to be redundant in their functions.

This work provides novel evidence in the field of neuronal differentiation, given that it was unknown that CREB3L2, commonly associated with ER stress, also participates in NGF-induced neuronal differentiation of PC12 cells. Further characterization of downstream targets of CREB3L2 and the other CREB3 family members is required to reveal their participation in axonal growth and neuritic processes, either in the context of development or regeneration.

## Data Availability Statement

The original contributions presented in the study are included in the article/Supplementary Material, further inquiries can be directed to the corresponding author.

## Author Contributions

LS, MFC, PDG, and CA conceived and designed the experiments. LS and MFC performed the experiments. LS, MFC, PDG, VR-S, and CA analyzed the data. PDG and VR-S contributed to reagents, materials, and analysis tools. LS, MFC, VR-S, and CA wrote the manuscript. All authors contributed to the article and approved the submitted version.

## Conflict of Interest

The authors declare that the research was conducted in the absence of any commercial or financial relationships that could be construed as a potential conflict of interest.

## Publisher’s Note

All claims expressed in this article are solely those of the authors and do not necessarily represent those of their affiliated organizations, or those of the publisher, the editors and the reviewers. Any product that may be evaluated in this article, or claim that may be made by its manufacturer, is not guaranteed or endorsed by the publisher.

## Supplementary Material

Supplementary Figure 1 NGF-differentiated PC12 cells. **(A)** Representative images of PC12 treated with NGF (100 ng/ml) during the indicated days (D2, D4, and D6). Untreated cells (D0) are considered as control. **(B)** Western blot assays performed with cell lysates obtained from PC12 cells differentiated with NGF during the indicated days. One representative blot is shown. The bar graph shows the densitometric quantification of the indicated proteins relative to the nuclear protein KAP1 (loading control). Values from untreated cells were set equal to 1. The quantification was carried out, using Image Studio analysis software. Bars represent the mean ± SEM from three independent experiments, which were analyzed, using ANOVA test, followed by Bonferroni multiple comparison post-test, considering statistically significant a value of *p* < 0.05 (^∗^*p* < 0.05; ^∗∗^*p* < 0.001). Scale bar: 20 μm.

Supplementary Figure 2 Different shRNAs, shCREB3L2 I, and shCREB3L2 II, elicit similar effects. Representative images of differentiated PC12 cells transfected with shScramble-EGFP or two independent shCREB3L2-EGFP for 48 h before NGF treatment. **(A)** Representative immunofluorescence staining of CREB3L2 in PC12 cells transfected with shScramble, shCREB3L2 I, or shCREB3L2 II (D3). Fire-LUTs are shown to clearly visualize IF levels. **(B)** Representative images of PC12 cells transduced with shScramble and shCREB3L2 II and fixed at the indicated day post NGF induction. **(C)** Quantification of the CREB3L2 fluorescence intensity normalized with the control condition (shScramble). **(D,E)** Quantification of the total neurite length and the longest neurite. Bars represent the mean ± SEM of three independent experiments carried out in triplicates, and a total of 40 cells were analyzed. Statistical data analysis was performed, using Student’s *T*-test, considering statistically significant a value of *p* < 0.05 (^∗^*p* < 0.05, ^∗∗^*p* < 0.01; ^∗∗∗^*p* < 0.001). Scale bar: 20 μm.

Supplementary Figure 3 Gain- and loss-of functions of both Rab1b and Rab5, proteins associated with membrane trafficking pathway, alter the neuronal differentiation of PC12 cells. **(A)** Representative images of differentiated PC12 cells transfected with control EGFP, Rab1b WT-EGFP, or Rab1b N121I-EGFP plasmids and fixed at 4 days post NGF induction. Insets show Rab1b distribution in the cell soma. Quantification of the total neurite length **(B)** and the longest neurite **(C)**. **(D)** Representative images of differentiated PC12 cells transfected with control EGFP, Rab5 WT-EGFP, or Rab5 S34N-EGFP plasmids and fixed at 4 days post NGF induction. Quantification of the total neurite length **(E)** and the longest neurite **(F)**. Bars represent the mean ± SEM of three independent experiments carried out in triplicates, and a total of 40 cells were analyzed. Statistical data analysis was performed, using Student’s *T*-test, considering statistically significant a value of *p* < 0.05 (^∗^*p* < 0.05, ^∗∗^*p* < 0.01; ^∗∗∗^*p* < 0.001). Scale bar: 20 μm.
