# Supplementary figures and images for: CREB3L2 Modulates Nerve Growth Factor-Induced Cell Differentiation
Source: Front Mol Neurosci. 2021 Aug 3;14:650338. doi: 10.3389/fnmol.2021.650338 (PMC8370844; doi:10.3389/fnmol.2021.650338)

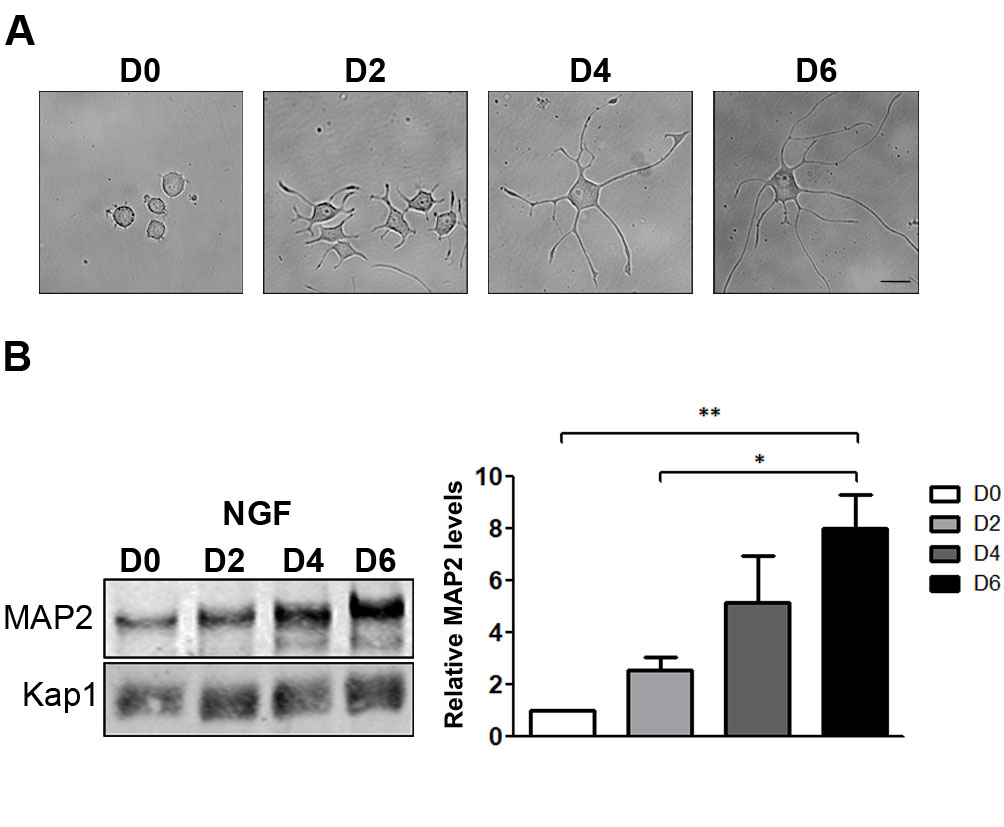

Supplement: Supplementary Figure 1 — NGF-differentiated PC12 cells. (A) Representative images of PC12 treated with NGF (100 ng/ml) during the indicated days (D2, D4, and D6). Untreated cells (D0) are considered as control. (B) Western blot assays performed with cell lysates obtained from PC12 cells differentiated with NGF during the indicated days. One representative blot is shown. The bar graph shows the densitometric quantification of the indicated proteins relative to the nuclear protein KAP1 (loading control). Values from untreated cells were set equal to 1. The quantification was carried out, using Image Studio analysis software. Bars represent the mean ± SEM from three independent experiments, which were analyzed, using ANOVA test, followed by Bonferroni multiple comparison post-test, considering statistically significant a value of p < 0.05 (∗p < 0.05; ∗∗p < 0.001). Scale bar: 20 μm. [file Image_1.JPEG]

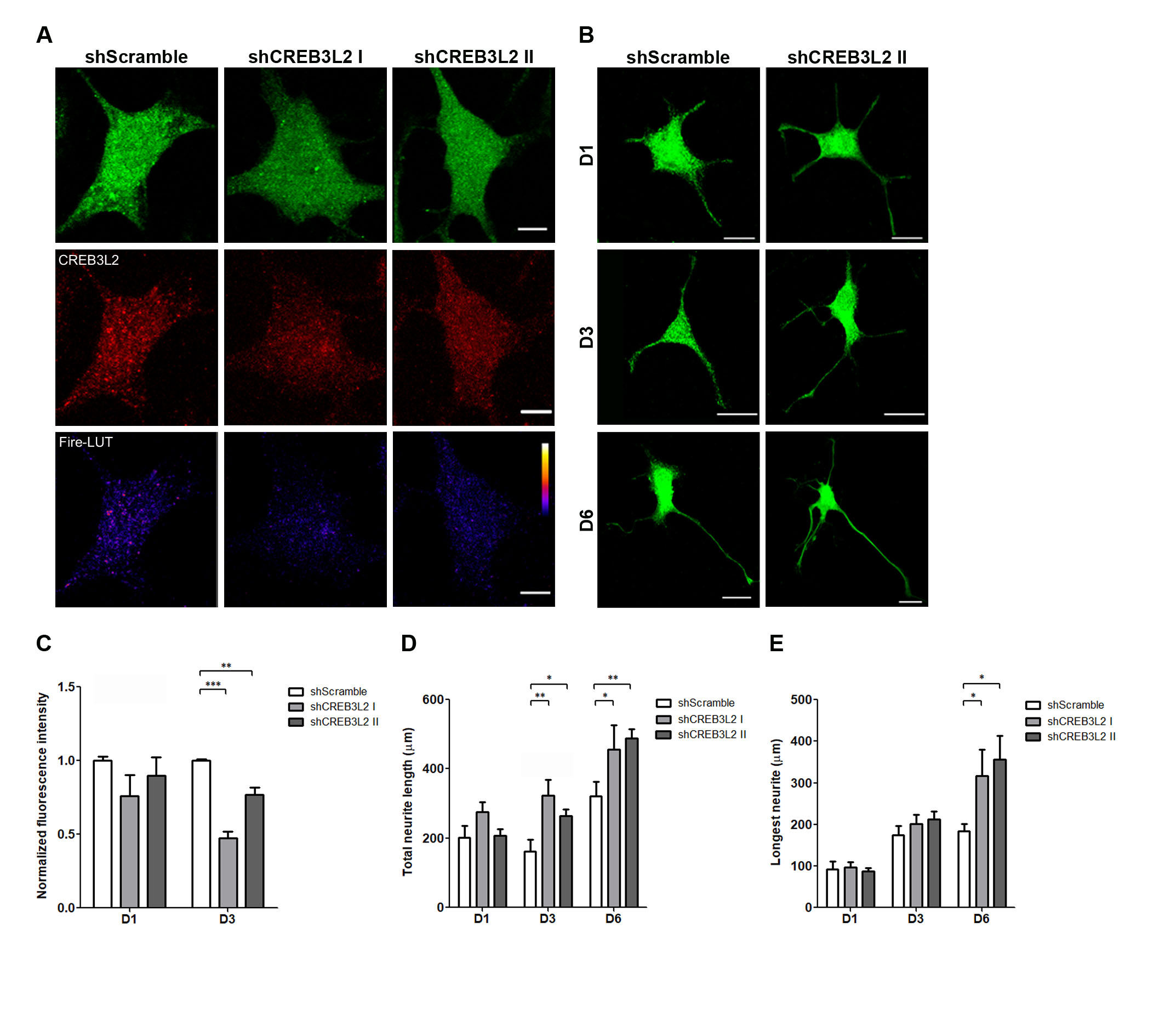

Supplement: Supplementary Figure 2 — Different shRNAs, shCREB3L2 I, and shCREB3L2 II, elicit similar effects. Representative images of differentiated PC12 cells transfected with shScramble-EGFP or two independent shCREB3L2-EGFP for 48 h before NGF treatment. (A) Representative immunofluorescence staining of CREB3L2 in PC12 cells transfected with shScramble, shCREB3L2 I, or shCREB3L2 II (D3). Fire-LUTs are shown to clearly visualize IF levels. (B) Representative images of PC12 cells transduced with shScramble and shCREB3L2 II and fixed at the indicated day post NGF induction. (C) Quantification of the CREB3L2 fluorescence intensity normalized with the control condition (shScramble). (D,E) Quantification of the total neurite length and the longest neurite. Bars represent the mean ± SEM of three independent experiments carried out in triplicates, and a total of 40 cells were analyzed. Statistical data analysis was performed, using Student’s T-test, considering statistically significant a value of p < 0.05 (∗p < 0.05, ∗∗p < 0.01; ∗∗∗p < 0.001). Scale bar: 20 μm. [file Image_2.JPEG]

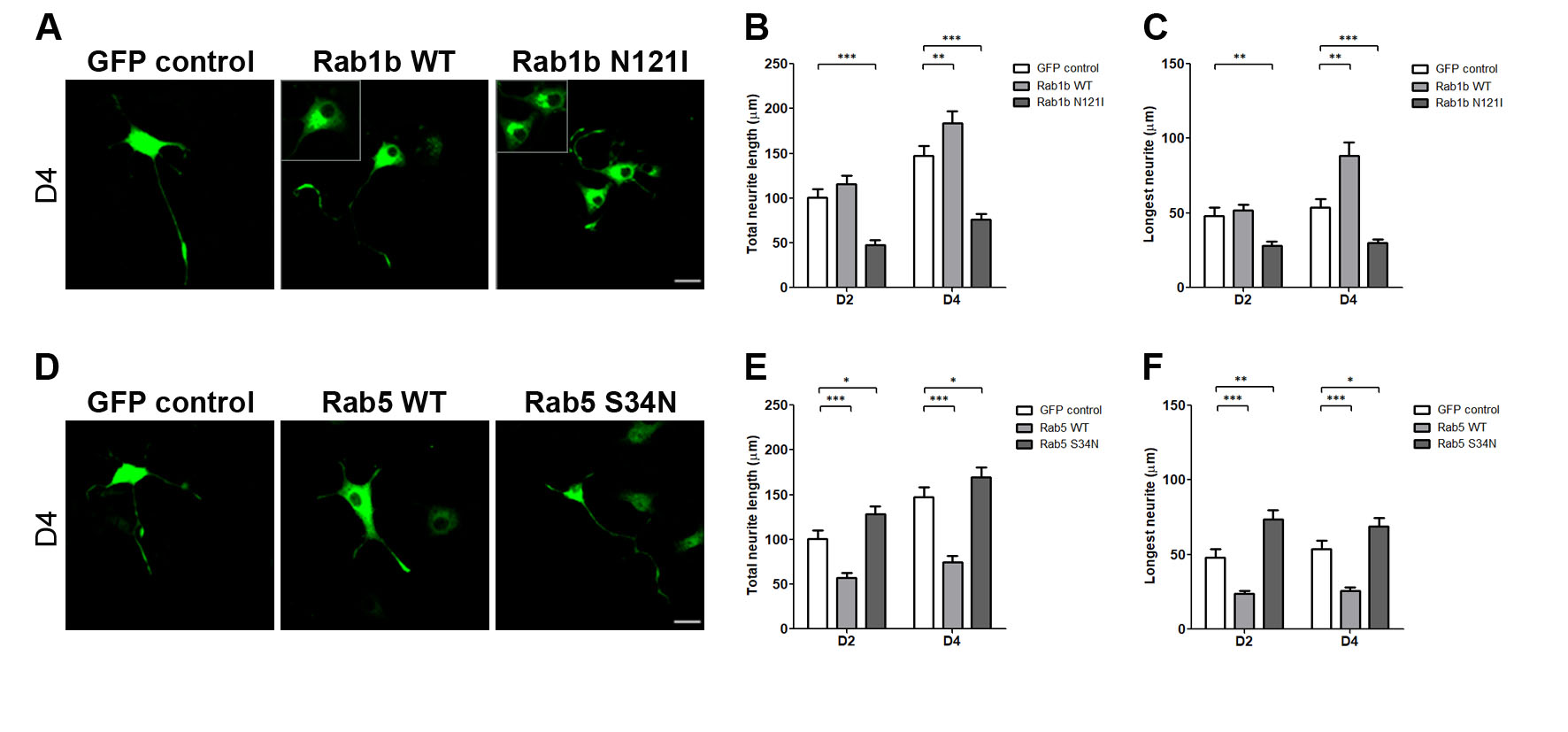

Supplement: Supplementary Figure 3 — Gain- and loss-of functions of both Rab1b and Rab5, proteins associated with membrane trafficking pathway, alter the neuronal differentiation of PC12 cells. (A) Representative images of differentiated PC12 cells transfected with control EGFP, Rab1b WT-EGFP, or Rab1b N121I-EGFP plasmids and fixed at 4 days post NGF induction. Insets show Rab1b distribution in the cell soma. Quantification of the total neurite length (B) and the longest neurite (C). (D) Representative images of differentiated PC12 cells transfected with control EGFP, Rab5 WT-EGFP, or Rab5 S34N-EGFP plasmids and fixed at 4 days post NGF induction. Quantification of the total neurite length (E) and the longest neurite (F). Bars represent the mean ± SEM of three independent experiments carried out in triplicates, and a total of 40 cells were analyzed. Statistical data analysis was performed, using Student’s T-test, considering statistically significant a value of p < 0.05 (∗p < 0.05, ∗∗p < 0.01; ∗∗∗p < 0.001). Scale bar: 20 μm. [file Image_3.JPEG]
